# Structural and Biochemical Properties of Duckweed Surface Cuticle

**DOI:** 10.3389/fchem.2018.00317

**Published:** 2018-07-26

**Authors:** Nikolai Borisjuk, Anton A. Peterson, Jiyang Lv, Guorun Qu, Qian Luo, Lei Shi, Guimin Chen, Olena Kishchenko, Yuzhen Zhou, Jianxin Shi

**Affiliations:** ^1^Jiangsu Key Laboratory for Eco-Agricultural Biotechnology around Hongze Lake, School of Life Science, Huaiyin Normal University, Huaian, China; ^2^Joint International Research Laboratory of Metabolic and Developmental Sciences, School of Life Sciences and Biotechnology, Shanghai Jiao Tong University, Shanghai, China

**Keywords:** duckweed, *Spirodela polyrhiza*, biodiversity, genotyping, cuticle wax, fatty acids, phytosterols

## Abstract

The plant cuticle, which consists of cutin and waxes, forms a hydrophobic coating covering the aerial surfaces of all plants. It acts as an interface between plants and their surrounding environment whilst also protecting them against biotic and abiotic stresses. In this research, we have investigated the biodiversity and cuticle properties of aquatic plant duckweed, using samples isolated from four different locations around Hongze lake in Jiangsu province, China. The samples were genotyped using two chloroplast markers and nuclear ribosomal DNA markers, which revealed them as ecotypes of the larger duckweed, *Spirodela polyrhiza*. Duckweed cuticle properties were investigated by compositional analysis using Gas Chromatography coupled with Mass Spectroscopy (GC-MS) Flame Ionization Detector (GC-FID), and ultrastructural observation by cryo-Scanning Electron Microscopy (cryo-SEM). Cuticle compositional analysis indicated that fatty acids and primary alcohols, the two typical constituents found in many land plant cuticle, are the major duckweed wax components. A large portion of the duckweed wax fraction is composed of phytosterols, represented by campesterol, stigmasterol, sitosterol and their common precursor squalene. The cryo-SEM observation uncovered significant differences between the surface structures of the top air-facing and bottom water-facing sides of the plant fronds. The top side of the fronds, containing multiple stomata complexes, appeared to be represented by a rather flat waxy film sporadically covered with wax crystals. Underneath the waxy film was detected a barely distinguished nanoridge net, which became distinctly noticeable after chloroform treatment. On the bottom side of the fronds, the large epidermal cells were covered by the well-structured net, whose sections became narrower and sharper under cryo-SEM following chloroform treatment. These structural differences between the abaxial and adaxial sides of the fronds evidently relate to their distinct physiological roles in interacting with the contrasting environments of sunlight/air and nutrients/water. The unique structural and biochemical features of *Spirodela* frond surfaces with their rapid reproductive cycle and readily availability genome sequence, make duckweed an attractive monocot model for studying the fundamental processes related to plant protection against ultraviolet irradiation, pathogens and other environmental stresses.

## Introduction

The plant cuticle, a skin of the plant, forms a hydrophobic coating that covers aerial surfaces of all plants. In conjunction with stomata, the cuticle acts as an interface between plants and their surrounding environment, protecting them against a variety of abiotic stresses and pathogens. The cuticle not only acts as a protective interface but also controls the diffusion of molecules, with the most important function of the cuticle in protecting the aerial parts of terrestrial plants against water loss (Samuels et al., 2008).

Plant cuticle is generally composed of a cutin matrix covered or/and embedded with layers of cuticular waxes, however considerable variation in cuticle composition exists between species, plant organs and developmental stages (Yeats and Rose, 2013). The typical cutin matrix is represented by hydroxy and/or epoxy C16/C18 fatty acids cross-linked by ester bonds into an elastic polyester structure (Fich et al., 2016; Bakan and Marion, 2017). The waxes consist of a combination of various aliphatic molecules, such as very long fatty acids (with a chain length of C20-C24), primary alcohols (C22-C40), alkanes (C21-C35), aldehydes (C24-C36), ketones (C21-C35), and diketones (C22-C36). These lipid derivatives can be embedded in the cutin matrix (intracuticular waxes), or deposited on the cuticle surface (epicuticular waxes). These aliphatic waxes define one of the essential features of primary plant surfaces, their hydrophobicity. This serves to repel water, other aqueous solutions and small organisms (Müller and Riederer, 2005). Epicuticular waxes deposited on the outer cuticle surface often form crystals of different shape visible under an electron microscope (Barthlott et al., 1998). In addition to waxes, some other components like phenolic represented by cinnamic acid or flavonoids could be also present in the cutin matrix (Domínguez et al., 2011).

The biochemical composition, as well as biosynthesis pathways leading to the accumulation of cuticle components, have been intensively studied in a number of terrestrial plants. Most intensively in the model plant Arabidopsis, tomato, maize and barley (Jetter et al., 2007; Bernard and Joubes, 2013; Borisjuk et al., 2014). As a result of these efforts, substantial progress has been made in recent decades for characterizing the biochemical and molecular mechanisms of cutin and wax synthesis and export (Samuels et al., 2008; Kunst and Samuels, 2009), cutin monomer synthesis and assembly (Beisson et al., 2012), cuticle involvement in biotic and abiotic stress responses (Bessire et al., 2007; Seo and Park, 2011) and regulation of organ development (Ingram and Nawrath, 2017). However, many aspects of cuticle biology remain unclear, especially for specialized groups of plants, such as aquatic inhabitants. While aquatic lotus, *Nelumbo nucifera*, has been intensively studied in respect of the unique hydrophobic properties of its leaf surface (Ensikat et al., 2011), not much is known about the specificity of cuticle surface structure and biochemical composition in relation to inhabiting water surfaces.

Duckweed is a group of monocot aquatic plants endemic to most parts of the world. Its productivity can reach 80–100 tons dry mass per hectare per year, over 5 times as high as maize (Lam et al., 2014). In the process of biomass accumulation, duckweeds can very efficiently remediate different types of wastewater (Ziegler et al., 2016; Zhou et al., 2018). These complementary features - water remediation and fast biomass accumulation have made duckweed an object of intense global research interest in recent years (Lam et al., 2014; Appenroth et al., 2015). This increased level of research has resulted in the genome sequencing of two representative species *Spirodela polyrhiza* and Lemna minor (Wang et al., 2014; Van Hoeck et al., 2015; Michael et al., 2017), the establishment of specialized international conferences and an active and diverse academic community studying duckweed biology.

For aquatic plants, such as duckweed, which have an unlimited supply of water through constant contact of leaf and/or roots with water, water conservation inside the tissues should not be as important as it is for terrestrial plants grown under limited water supply. Instead, since duckweed has no option (mechanism) to protectively adjust frond position relative to sunlight, as terrestrial plants can do turning their leaves and flowers to an optimal position relative to the sun, the duckweed's frond surface should provide a sustainable protection against potentially harmful ultraviolet (UV) radiation. Protection against UV has been attributed mainly to the light scattering by the surface cuticular waxes (Long et al., 2003) and UV attenuation properties of various phenolic compounds incorporated into cuticle matrix (Rozema et al., 2002; Chen et al., 2013).

The second important function of the cuticle in aquatic plants concerns protection against pathogens, both airborne and those inhabiting the water environment. The interaction of aerial surfaces of land plants with microbial pathogens and insects has been intensively investigated, especially in the model plant Arabidopsis and some agriculturally important cereals, (Reina-Pinto and Yephremov, 2009; Serrano et al., 2014; Kumar et al., 2016). However, almost no studies exist on the role of cuticles in aquatic plant-pathogen interaction at water surfaces.

As duckweed is becoming a popular prospective source of biomass, its cuticles also define the plants nutrient quality and other industrial applications (Petit et al., 2017). In this study, for the first time we present data on the surface structure (SEM) and biochemical composition (GC-MS/GC-FID) of cuticle in great duckweed, *Spirodella polyrhiza*, a duckweed species with a great potential for wastewater remediation (Ziegler et al., 2016), production of biofuel and animals/fish feed (Cheng and Stomp, 2009).

## Materials and methods

### Plant material

Four ecotypes of duckweed used in this study were collected at different locations around Hongze lake in Jiangsu province, China, in October 2016. The GPS coordinates for the samples collection are: N 33″38′41/E 118″58′33 for sample G (RDSC clone registration: 5545); sample K: N 33″22′43; E 118″53′23 (RDSC clone registration: 5546); sample M: N 33″19′41; E 118″51′43 (RDSC clone registration: 5547); sample N: N 33″17′40; E 118″49′45 (RDSC clone registration: 5548).

### Identity recognition

Prior to further examinations, duckweed samples were washed with water and cultivated on the surface of sterilized SH medium for 2 weeks. The identity of the collected duckweed ecotypes was examined by DNA barcoding using primers specific for chloroplast intergenic spacers *atpF-atpH* (ATP) and *psbK-psbL* (PSB) as previously described (Borisjuk et al., 2015). Additionally, the biodiversity of the collected duckweed strains was estimated by comparison of DNA sequences of intergenic spacers on the nuclear 5S ribosomal genes (rDNA). The DNA fragments of 5S rDNA spacers were amplified by PCR using 5S gene-specific primers DW-5S-F: CTTGGGCGAGAGTAGTACTAGG and DW-5S-R: CACGCTTAACTTCGGAGTTCTG, purified by gel electrophoresis and sequenced using the DW-5S-F primer. The obtained sequences were aligned using the “Online Analysis Tools” package (http://molbiol-tools.ca).

### Microscopic observation of duckweed surfaces

The air and water-facing surfaces of duckweed fronds and turions were examined using a cryo-scanning electron microscope (cryo-SEM, Hitachi S3400II). The fresh duckweed fronds and turions were carefully mounted on the copper stage with glue, immediately frozen with liquid nitrogen, sprayed with gold, and observed under low vacuum mode. Images were taken using its carrying camera.

The autofluorescence of duckweed surfaces was monitored using fronds decolorized by repeated incubation in 70% ethyl alcohol (Vitha et al., 1995) supplemented with 10% sucrose at 37°C. Following decolorization, a half of the fronds were incubated overnight at 37°C in 0.5M NaOH in order to remove cell wall bound phenolic acids, a potential source of autofluorescence (Ride and Pearce, 1979). The discolored fronds were sliced using LEICA CM1850 cryomicrotome. The autofluorescence of the resulted 20 μm slices, was observed using LEICA DM2500 fluorescent microscope equipped with 360 and 480 nm wavelength excitation filters. The phloroglucinol-HCl staining was performed following the protocol described by Donaldson and Williams (2018) with some modifications.

### Identification and quantification of duckweed waxes and cutin monomers

For wax analyses, lyophilized or fresh duckweed fronds were immersed in 2 ml chloroform using two time regimes. First, the fronds were treated with chloroform for 60 s twice and the two extracts were pooled; second, the fronds were treated with chloroform for 30 s. Following wax extraction, 50 μl of internal standard (C24 alkane; 10 mg/ 50 ml) was added. Extracts with internal standard were dried under a stream of nitrogen to a final volume of 100 μl. Next, 20 μl pyridine and 20 μl BSTFA [*N*,*O*-bis(trimethylsilyl) trifluoroacetamide] were added and the mixtures were incubated at 70°C for 40 min. Wax solutions were transferred to GC vials and analyzed by Gas Chromatography-Mass Spectrometry/Gas Chromatography-Flame Ionization Detector (GC-MS/GC-FID) as described by Zhu et al. (2013). For cutin analysis, samples that had been used in the wax extraction were exhaustedly extracted with chloroform/methanol (1:1 v/v) for 2 weeks with daily changing in the solvent. The remaining delipidated samples were transesterified with 1 mL of 1 N methanolic HCl for 2 h at 80°C. After the addition of 2 ml of saturated NaCl and 20 mg of dotriacontane (Fluka) as an internal standard, the hydrophobic monomers were subsequently extracted three times with 1 ml of hexane. The organic phases were combined, evaporated, and derivatized as described above. The GC-MS and GC-FID analysis were performed using a procedure identical to that of the wax analysis. Results were calculated as per unit surface area.

### Statistical analysis

Not less than four biological replicas were analyzed by GS-MS for each duckweed ecotypes. For each of the identified individual chemical substances the mean values and standard deviations were calculated. Surface areas of fronds samples were measured using ImageJ graphical software (https://imagej.nih.gov/ij/). Percentages of the individual substances in cuticle waxes and individual monomers in depolymerized cutin fraction were calculated using data of individual substances mean content in ug/cm^2^ of duckweed fronds surface.

One-way multivariate analysis of variance (MANOVA) was performed using General Linear Model Multivariate analysis module of the “SPSS Statistics” (IBM) software. Statistical significances of effects of ecotypes variations on the values of individual substance content in wax and cutin were calculated using *Post Hoc* Multiple Comparisons Tukey's Tests. The results of this test include grouping of ecotypes into homogeneous subsets according to the calculated statistical significances of differences in each substance mean content.

Representative cryo SEM images were chosen from not less than 30 micrographs of individual fronds or turions of each ecotype.

## Results

### The identity of the duckweed strains

**Figure 1:**
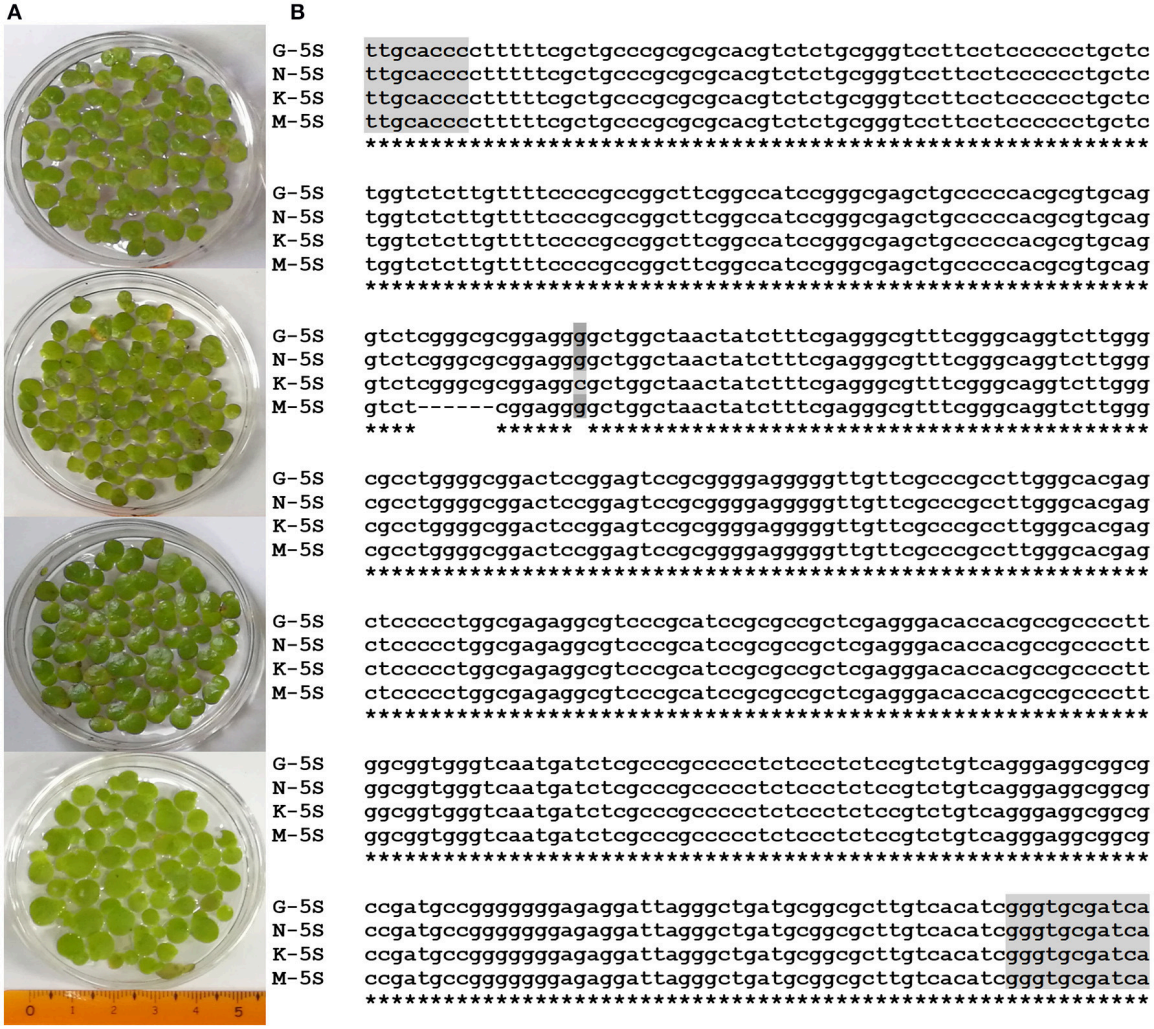
Images of *S. polyrhiza* frond ecotypes G, K, M, and N **(A)**, and sequence alignment of their 5S rDNA spacers **(B)**. Parts of sequences encoding 5S ribosomal RNA are highlighted by gray.

Four duckweed strains collected around Hongze lake, used in the experiments (Figure 1), were genotyped using dual chloroplast DNA barcodes (Borisjuk et al., 2015), atpF-atpH (ATP) and psbK-psbL (PSB) spacer sequences, and nuclear 5S ribosomal DNA (rDNA) spacer. Chloroplast DNA fragments, amplified by PCR, were directly sequenced using one of the amplification primers and blasted against the sequences available in the NCBI DNA database. Both ATP and PSB clearly identified the samples as large duckweed, *Spirodela polyrhiza* (Appenroth et al., 2013). The PSB sequences show no variations between the four *Spirodela* strains, while the ATP demonstrated some sequence variations between the isolates, with most of them being G/A substitutions (Supplement Figure 1). Sequence alignments of the 5S rDNA spacer, only allowed analysis of the variations between the isolates, as no similar sequences were found in the DNA database. The alignment revealed a short 6 nucleotide deletion in strain M, and a G to C substitution in strain K (Figure 1B).

### Cryo-SEM images of duckweed cuticle surface

**Figure 2:**
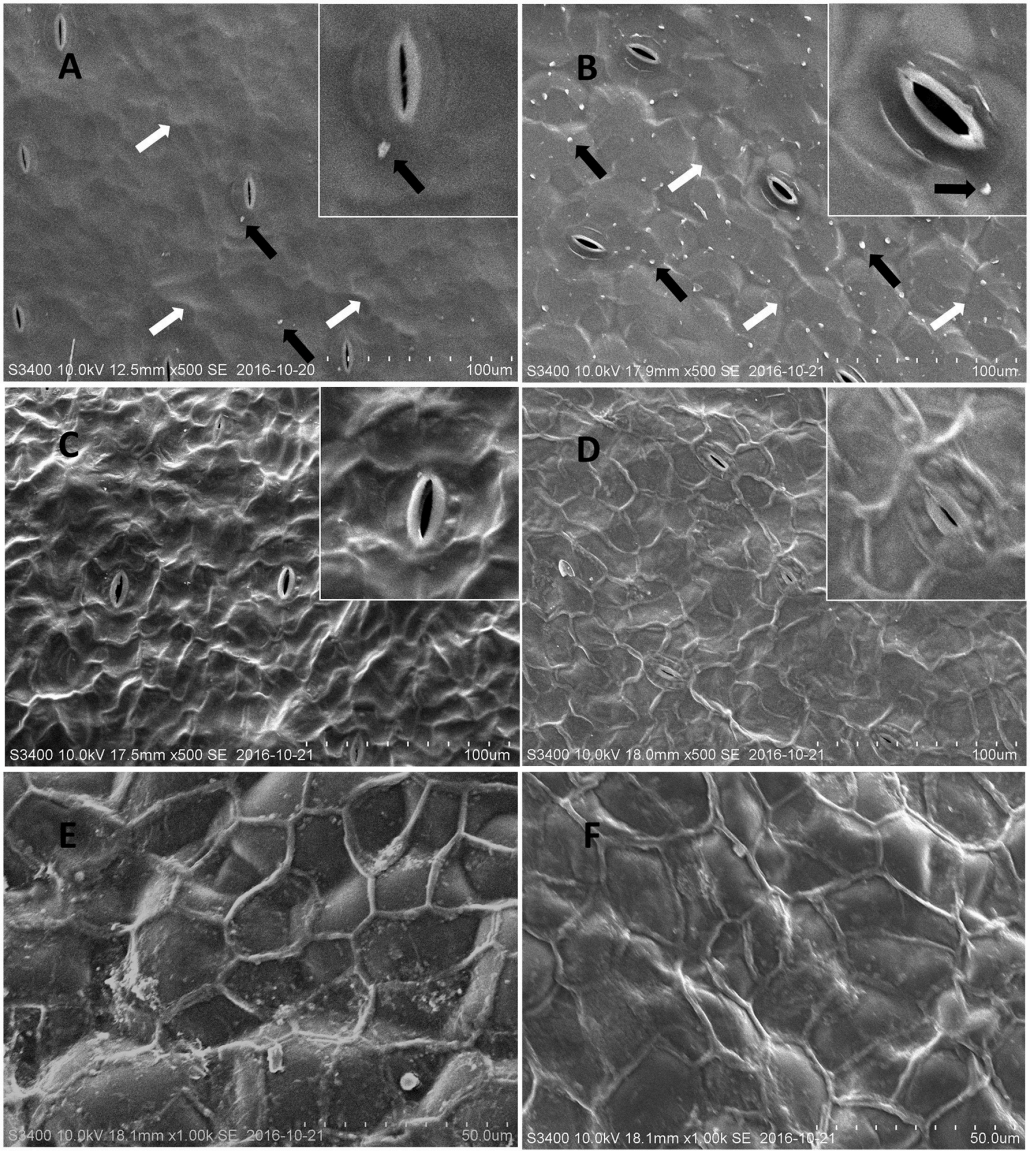
Representative cryo-SEM micrographs of *S. polyrhiza* frond surfaces. Images of the intact adaxial (air-facing) side of *S. polyrhiza* fronds from strain M **(A)** and strain N **(B)**. Images of the adaxial side of *S. polyrhiza* fronds following treatment with chloroform from strain M **(C)** and strain N **(D)**. Images of the intact **(E)** and chloroform treated **(F)** abaxial (water-facing) side of *S. polyrhiza* frond from strain M. White arrows indicate unevenness in areas of epidermal cells joints and black arrows indicate wax crystalloids.

To investigate the structural features of the frond and turion surfaces we have performed cryo-SEM analysis, focusing on both intact surfaces of duckweed fronds and turions and the frond surfaces treated with chloroform (to remove epicuticular waxes). The air-facing and water-facing surfaces of all four isolates of *S. polyrhiza* were examined and the representative images of ecotypes N and M are presented in Figure 2.

The intact adaxial, air-facing side of frond cuticle of the investigated duckweed ecotypes, appeared as a predominantly smooth film spread with distinct stomata and some irregular unevenness, covering most of the morphology of underlying epidermal cells (Figures 2A,B). On the surface of this film, solitary wax crystalloids in the form of “granules” could be observed to be sparsely deposited as proposed by Barthlott et al. (1998) (Figures 2A,B). It was noted that ecotype N (Figure 2B) has a higher density of surface wax granules deposition, compared to ecotype M (Figure 2A). After being treated with chloroform, the wax “granules” and the waxy film were removed and this chloroform treatment transformed the original smooth waxy surface into the net-like nanoridge surface (Figures 2C,D).

The abaxial, water-facing side of the fronds showed a significantly different surface structure from the adaxial side. It did not have stomata (Figure 2E) but had well organized large oval cells (with the size of about 40 μm) fully covered with a thick waxy film, on which an extracellular net (with a single section diameter of 20–30 μm) formed. Upon chloroform treatment, the waxy film was removed, while the extracellular nanoridge became thinner and the topography of epidermal cells appeared, which seemed to have lost their rigidity (Figure 2F).

**Figure 3:**
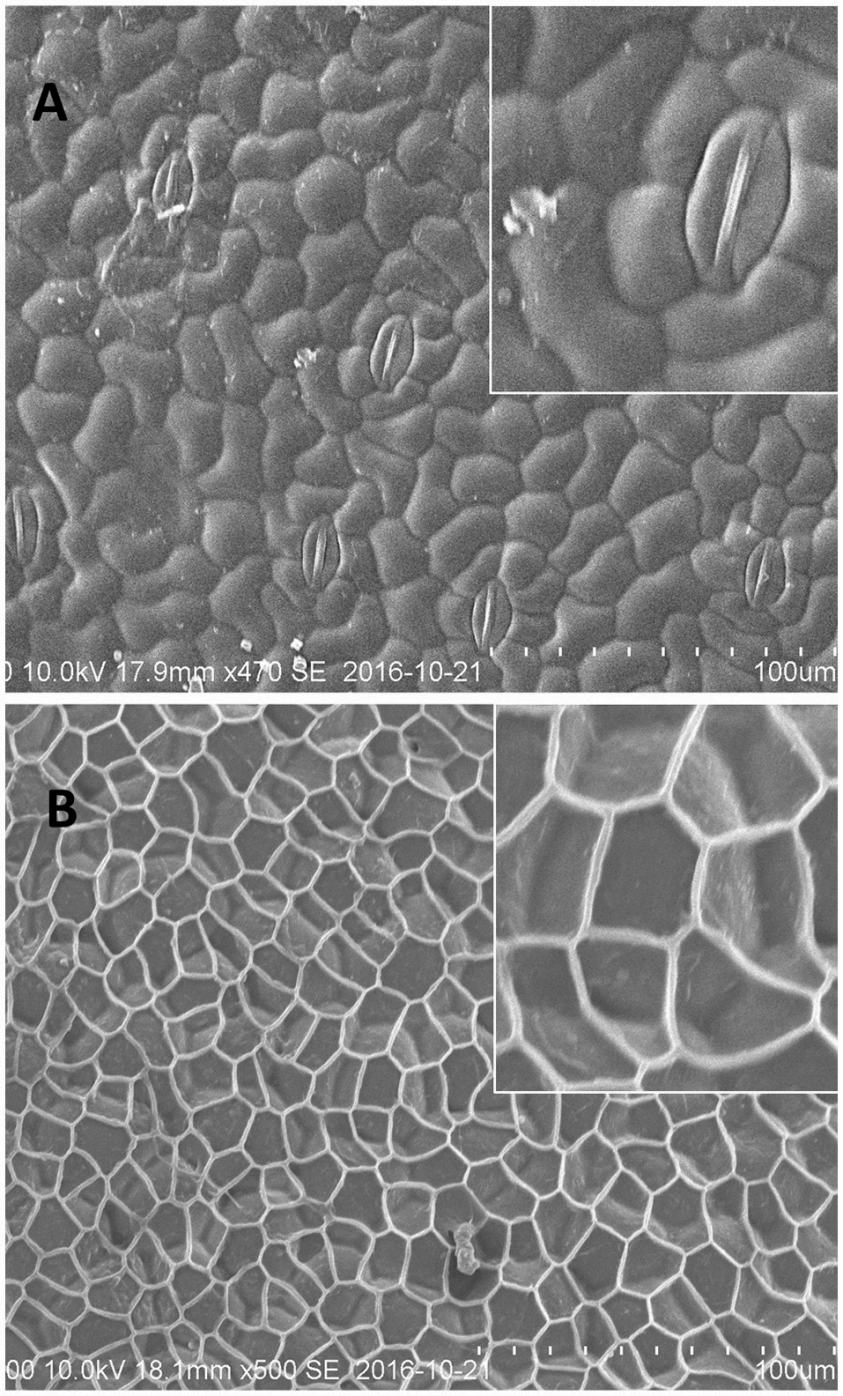
Representative cryo-SEM micrographs of *S. polyrhiza* turion surfaces. **(A)** Adaxial side of the turion with stomatal complexes. **(B)** Abaxial side of the turion.

Similar to fronds, the *Spirodela* turions, displayed a very distinct surface structure of abaxial and adaxial sides (Figure 3). The abaxial turion side, defined by the absence of stomata, had a structure to a large extent resembling that of the frond's abaxial side (similar size of cells, ~40 μm and sections of net, ~20–30 μm), with a more profoundly expressed extracellular net (Figure 3B). Whereas, the adaxial side of turions, was distinguished by well-defined stomata complexes with closed stomata and did not have the similar smooth waxy surface appearance to that of the frond's abaxial side. The contours of the epidermal cells, with the size of about 20 μm, were very well visible with the clearly defined borders between them. Few structures, similar to wax “granules” on the fronds surface could be detected on the dense cell surfaces.

### Composition of the surface waxes of fronds and turions of *Spirodela polyrhiza*

**Figure 4:**
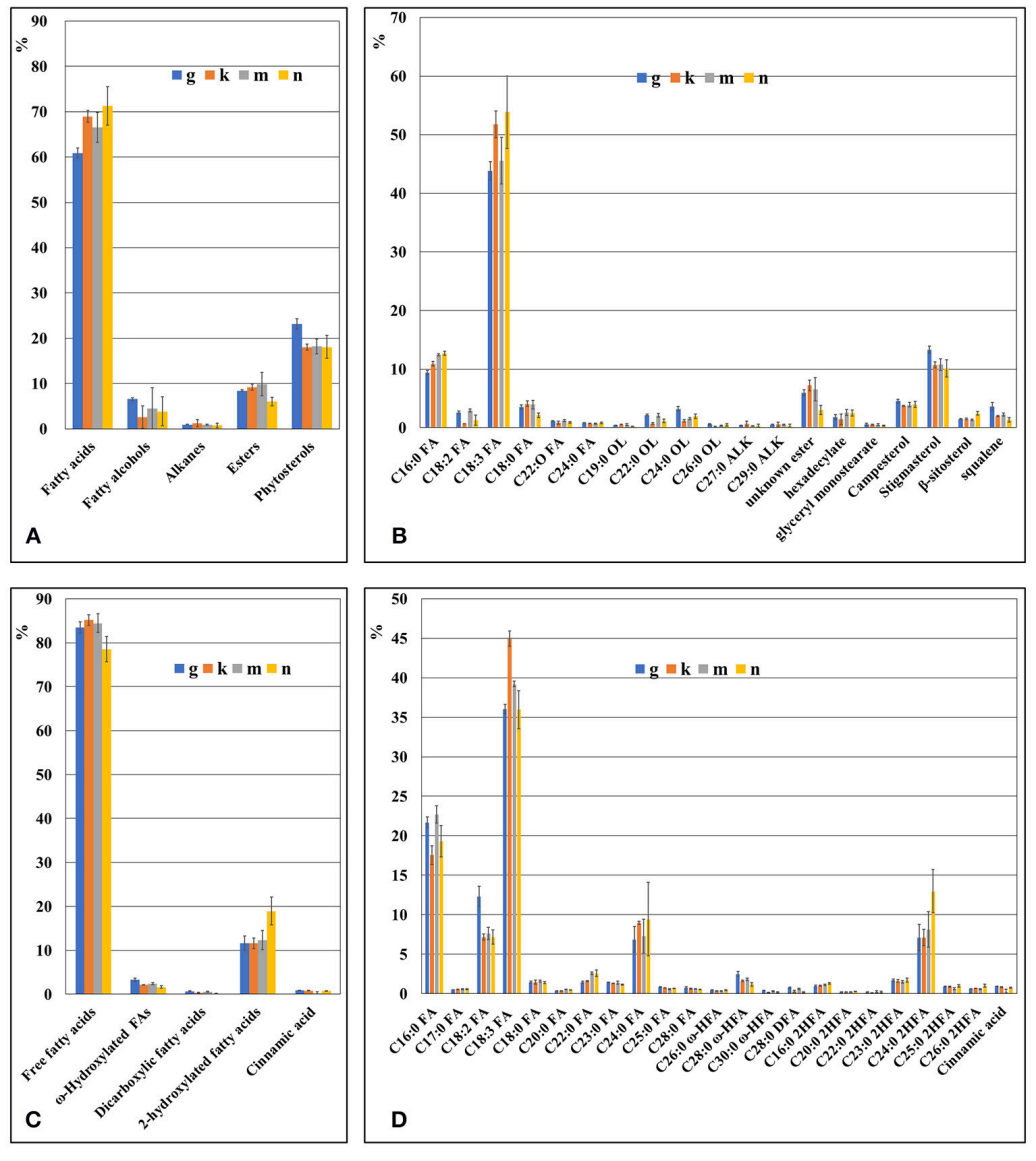
The chemical composition of duckweed fronds cuticle as revealed by GS-MS and GS-FID analysis. **(A)** Components percentage in wax fraction of the duckweed fronds surface. **(B)** Percentage of individual components in the wax fraction of duckweed fronds surface. **(C)** Components percentage in the cutin fraction of duckweed fronds. **(D)** Percentage of individual monomer components in the cutin fraction of duckweed fronds surface. C16:0 FA, hexadecanoic acid; C18:2 FA, (9Z,12Z)-octadeca-9,12-dienoic acid; C18:3 FA, octadecatrienoic acid; C18:0 FA, octadecanoic acid; C20:0 FA, icosanoic acid; C22:0 FA, docosanoic acid; C23:0 FA, tricosanoic acid; C24:0 FA, tetracosanoic acid; C25:0 FA, pentacosanoic acid; C28:0 FA, octacosanoic acid; C19:0 OL, nonadecan-1-ol; C22:0 OL, docosan-1-ol; C24:0 OL, tetracosan-1-ol; C26:0 OL, hexacosan-1-ol; C27:0 ALK, heptacosane; C29:0 ALK, nonacosane; C26:0 ω HFA, ω-hydroxy hexacosanoic acid; C28:0 ω-HFA, ω-hydroxy octacosanoic acid; C30:0 ω-HFA, ω-hydroxy triacontanoic acid; C28:0 DFA, octacosanedioic acid; C16:0 2HFA, dihydroxy hexadecanoic acid; C20:0 2HFA, dihydroxy icosanoic acid; C22:0 2HFA, dihydroxy docosanoic acid; C23:0 2HFA, dihydroxy tricosanoic acid; C24:0 2HFA, dihydroxy tetracosanoic acid; C25:0 2HFA, dihydroxy pentacosanoic acid; C26:0 2HFA, dihydroxy hexacosanoic acid.

The composition of waxes on the fronds and turions of four ecotypes (G, K, M, and N) of *Spirodela polyrhiza* was qualitatively and quantitatively analyzed using GC-MS and GC-FID, respectively. The wax fraction extracted from the fronds for 2 minute resulted in a mixture of free fatty acids, esters, fatty alcohols, alkanes, and phytosterols. Their compositions are shown in Figure 4A. Surprisingly, free fatty acids dominated, accounting for approximately 60 to 70% of the total soluble fraction; the next most common was phytosterols, accounting for about 20% of the total. Composition details for the major compounds is presented in Figure 4B. Repeating the procedure with an extraction time of 0.5 min revealed a similar pattern in major components, but the amounts differed. The percentage of free fatty acids became 18.8%, replaced by phytosterols, which increased to about 60% (Supplement Figure 2). The free fatty acids included both saturated and unsaturated types, ranging from 16 to 24 carbon atoms in length. When extracted for the longer period, the major individual fatty acids were saturated C16:0 (palmitic acid) and unsaturated C18:3 (α-linolenic acid), which is rather characteristic for intracellular membrane lipids, but at the shorter period, they were common cuticular waxes, saturated C16:0, C18:0, and C24:0.

Among the phytosterols was stigmasterol, campersterol, squalene, and β-sitosterol, the first representing the major component.

The saturated alcohols ranged in carbon chain length from 20 to 26, but they were predominantly 22 to 24 atoms in length, no matter the extraction time.

**Figure 5:**
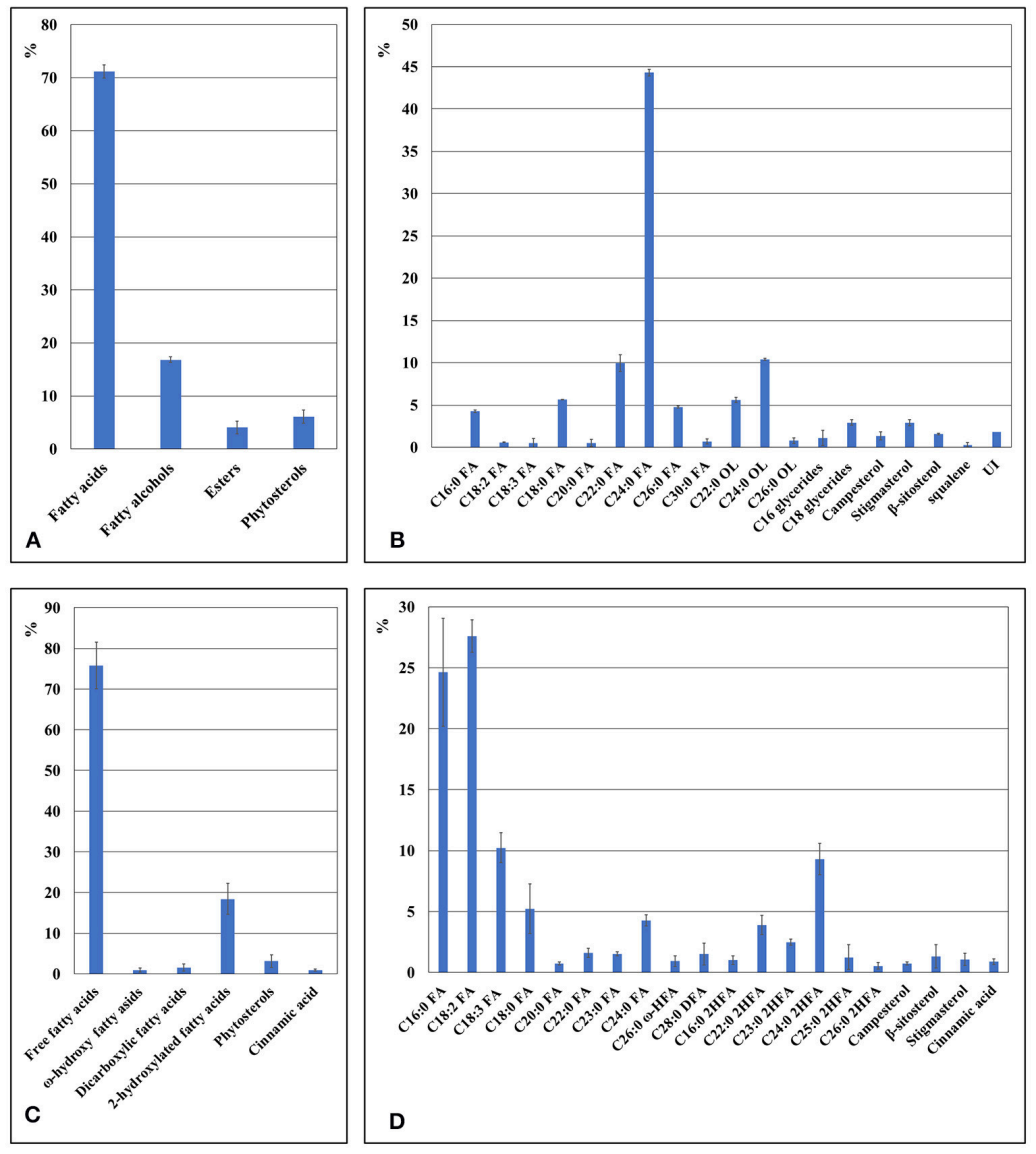
The chemical composition of cuticle in duckweed turions cuticle as revealed by GS-MS and GS-FID analysis. **(A)** Components percentage in wax fraction of the duckweed turions surface. **(B)** Percentage of individual components in the wax fraction of duckweed turions surface. **(C)** Components percentage in the cutin fraction of duckweed turions. **(D)** Percentage of individual monomer components in the cutin fraction of duckweed turions surface. C16:0 FA, hexadecanoic acid; C18:2 FA, (9Z,12Z)-octadeca-9,12-dienoic acid; C18:3 FA, octadecatrienoic acid; C18:0 FA, octadecanoic acid; C20:0 FA, icosanoic acid; C22:0 FA, docosanoic acid; C23:0 FA, tricosanoic acid; C24:0 FA, tetracosanoic acid; C26:0 FA, hexacosanoic acid; C30:0 FA, triacontanoic acid; C19:0 OL, nonadecan-1-ol; C22:0 OL, docosan-1-ol; C24:0 OL, tetracosan-1-ol; C26:0 OL, hexacosan-1-ol; C26:0 ω-HFA, ω-hydroxy hexacosanoic acid; C28:0 ω-HFA, ω-hydroxy octacosanoic acid; C30:0 ω-HFA, ω-hydroxy triacontanoic acid; C28:0 DFA, octacosanedioic acid; C16:0 2HFA, dihydroxy hexadecanoic acid; C22:0 2HFA, dihydroxy docosanoic acid; C23:0 2HFA, dihydroxy tricosanoic acid; C24:0 2HFA, dihydroxy tetracosanoic acid; C25:0 2HFA, dihydroxy pentacosanoic acid; C26:0 2HFA, dihydroxy hexacosanoic acid; UI(VE)-UI, unidentified substances.

The wax composition of turions was analyzed for *S. polyrhiza* acotype N, which was also used to characterize the turion surface by SEM (Figure 3). The major component of this wax fraction is fatty acids clearly dominated by saturated C24:0 (Figure 5). The wax of turions also contains saturated fatty alcohols, predominantly those 22 to 24 carbon atoms in length, and esters and phytosterols in lesser amounts.

### Biochemical analysis of cutin in duckweed fronds and turions

The cutin monomers found in duckweed fronds included several subgroups of fatty acids and aromatic acids as well as one with a phenolic ring–cinnamic acid (Figure 4C). In total, the fatty acids comprised more than 95% of the cutin monomers. Composition details of the major individual monomers are presented in Figure 4D.

The major individual fatty acids were saturated C16:0 (palmitic acid), comprising about 20% of the total soluble waxes, saturated C24:0, saturated 2-hydroxylated C24:0 acids, and unsaturated C18:2 (linoleic acid), each comprising about 8% of the total, and unsaturated C18:3, comprising about 35% of the total. The pattern of cutin monomers in the fronds was similar to that found in the cutin extracted from turions of ecotype N, but cutin from the turions contain a significantly higher percentage of C18:2 and less C18:3 compared to the fronds (Figures 5C, D).

**Figure 6:**
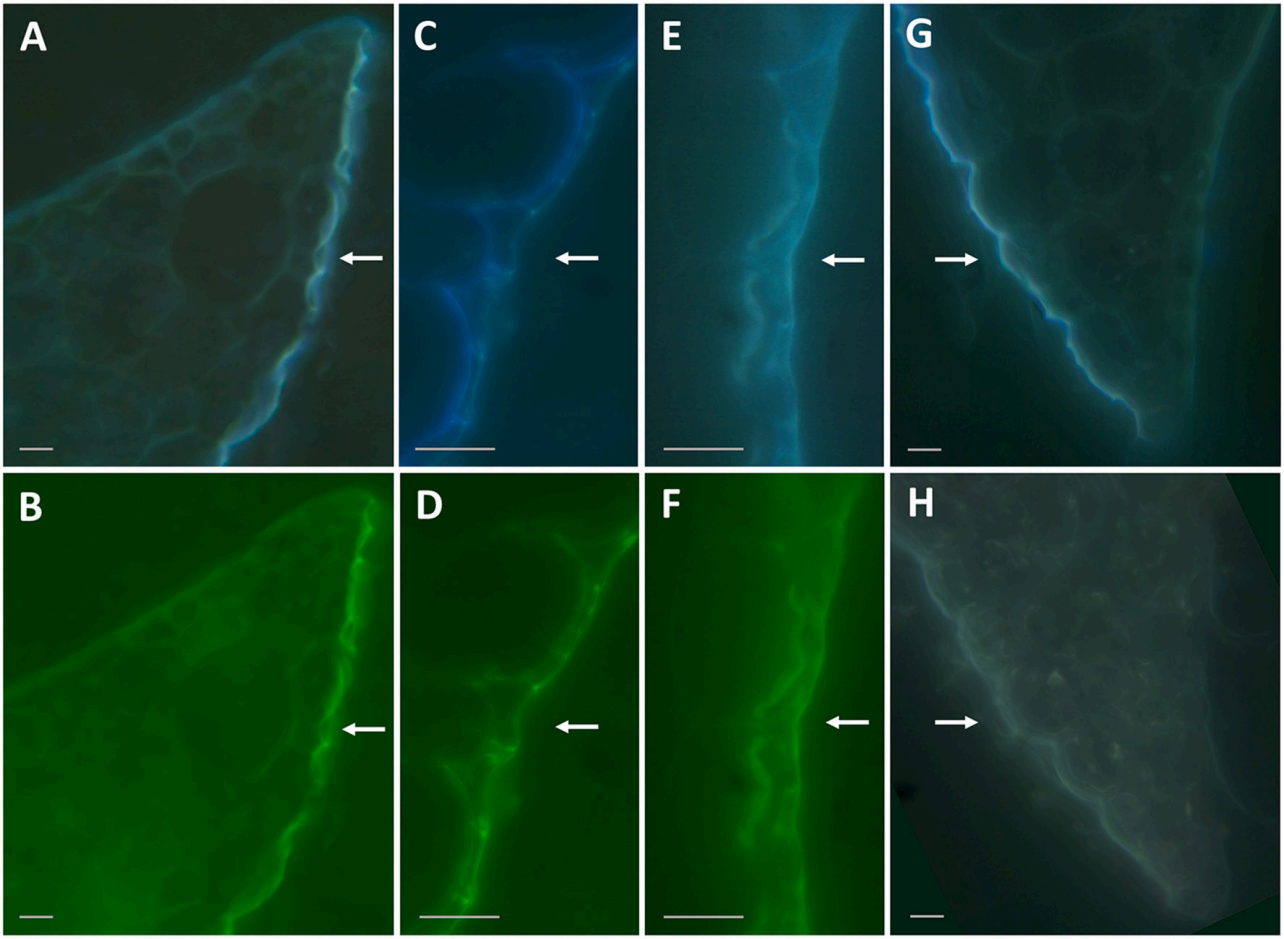
Autofluorescence of duckweed frond sections under 360 nm and 480 nm excitation. **(A,B)**: Representative images of cross-section of the frond edge with autofluorescence excited by 360 nm **(A)** and 480 nm **(B)** wavelength light. **(C,D)**: Same as **(A)** and **(B)** under higher magnification. **(E**,**F)**: Representative images of cross-sections of the fronds treated with 0.5 M NaOH and excited by 360 nm **(E)** and 480 nm **(F)** wavelength light. **(G,H)**: Representative images of cross-sections of the fronds excited by 360 nm wavelength light without any treatment **(G)** and following a treatment with phloroglucinol-HCl **(H)**. Bars correspond to 10 μm on the images. Arrows point to the water-facing side of duckweed fronds.

Because the presence of cinnamic acid, a common aromatic monomer of the polyaromatic domain of suberin and lignin (Bernards et al., 1995), could indicate suberinized layers in the duckweed epidermal cell walls, we investigated this possibility by performing autofluorescence tests on the ethanol-decolorized fronds. This treatment also removes flavonoids (Ferreira and Pinlio, 2012), the primary fluorescent agents in plant cuticle (Donaldson and Williams, 2018). As previously shown (Donaldson and Williams, 2018), suberin-like and lignin-like substances exhibit blue autofluorescence when excited by UV-A light (360 nm), and lignin-like substances exhibit green autofluorescence when excited by blue light (480 nm). At 360 nm and 480 nm, excitation revealed strong blue and green fluorescent signals on the water-facing sides of fronds (Figures 6A-D). Subsequently, a phloroglucinol staining test was performed, wherein the binding of phloroglucinol to polyphenol domains in lignin and suberin leads to autofluorescence quenching (Biggs, 1984). Figure 6H shows that treatment with phloroglucinol results in nearly complete absence of fluorescent signals compared to a non-treated slice (Figure 6G), supporting the presence of the lignin- and/or suberin-like polyaromatic domains at the water-facing surface of duckweed fronds. Figures 6E,F show autofluorescence of the water-facing surfaces following NaOH treatment, which removes cell-wall-bound phenolic acids (Ride and Pearce, 1979), which can be another source of autofluorescence (Biggs, 1984). Almost no difference was found between the blue and green fluorescent signals of treated (Figures 6E,F) and untreated samples (Figures 6C,D), further suggesting that the major fluorescent signals did not originate from cell-wall-bound phenolic acids, but rather lignin- and/or suberin-like compounds.

## Discussion

The cuticle, a continuous protective skin that covers all aerial surfaces of plants and serves as the interface between plant tissues and the environment, has been investigated in many aspects for a number of plant species and organs and at various developmental stages (Kunst and Samuels, 2009; De Luca and Valacchi, 2010; Ingram and Nawrath, 2017). Cutin and cuticular waxes composed of various long-chain (C20–C40) fatty acid derivatives (primary and secondary alcohols, alkanes, aldehydes, and esters) are recognized as the primary cuticle components (Domínguez et al., 2011). Within those components, a considerable variability in biochemical composition is found based on plant species (Jetter et al., 2007). This variability is primarily defined by the variant combination of basic aliphatic components and the incorporation of additional compounds such as terpenoids, flavonoids, and phenolic lipids (Hunt and Baker, 1980). Moreover, cuticles of particular plant groups contain taxon-specific components. For example, grasses are characterized by significant amounts of ß-diketones and related compounds (von Wettstein-Knowles, 2012; Bi et al., 2017). Current knowledge of cuticle structure and function originates almost exclusively from studies of terrestrial plants, for which the primary role of the cuticle is protection against water loss.

However, a substantial portion of species, the aquatic plants (or hydrophytes), inhabit water-based environments such as ponds, lakes, and other small reservoirs. Because of their unlimited water supply, protection against water loss is not as relevant for this group of flora. However, because aquatic floating-leaf plants adapted to growth on the water surface in a fixed position, they have no mechanism to protectively adjust their leaves and other organs relative to sunlight. Therefore, the surfaces of hydrophytes such as duckweed should function as effective protectors against potentially damaging UV irradiation. This is especially true in tropical and subtropical regions such as south China, where increased amounts of solar radiation are found.

In this study, we investigated the properties of the duckweed cuticle in four ecotypes grown around Hongze Lake in Jiangsu province, China by compositional analysis using GC-MS/GC-FID and cryo-SEM. Samples isolated from four locations were genotyped using two chloroplast markers and nuclear ribosomal DNA markers. The chloroplast DNA markers identified the samples as the greater duckweed, *Spirodela polyrhiza*, a dominant duckweed species in southern and middle China (Tang et al., 2015). The chloroplast ATP barcode showed some variability between samples, characteristic for the sequence variation between ecotypes of the same species (Supplement Figure 1). Similar variations were detected in nuclear 5S rDNA spacer sequences (Figure 1B). Correspondingly, compositional analysis of the wax and cutin cuticle components showed curtain levels of variations between ecotypes (Figure 4). Statistical analysis using the GLM multivariate test confirms that the majority of the wax and cutin components found in the four duckweed isolates can be quantitatively grouped within unique homogeneous domains (Supplement Tables 1, 2).

Chemical analyses of chloroform-extracted cuticle surface waxes revealed fatty acids, primary alcohols, esters, and alkanes (Figure 4A), typical constituents found in the cuticles of terrestrial plants, as the major cuticle components in *S. polyrhiza*. However, based on two extraction times, results show that the proportions of these components depend on the contact time between the fronds and the chloroform extractant. At the longer extraction time, the most prevalent component was the various types of free fatty acids (65%), followed by phytosterols (20%) dominated by C18:3 (linolenic acid) and C16:0 (palmitic acid; Figures 4A, B). At the shorter extraction time, phytosterols were the primary components in the fronds (about 60%), and the fatty acids followed in abundance (18.8%), dominated by saturated C16:0, C18:0, and C24:0. Such a difference in the proportion of fatty acids most probably reflects the fragile nature of *S. polyrhiza* fronds, compared to other plants, such as wheat (Bi et al., 2016), and the high proportion of fatty acids in the samples extracted for longer time most probably resulted from lipids leaking from internal tissues. Therefore, additional tests using the careful enzyme-assisted separation of cuticle (Guzman et al., 2014) are needed to gain a more accurate estimation of cuticle components.

Phytosterols have been identified in surface extracts of many plants (Jetter et al., 2007), however the high percentage (up to 60%) of phytosterols represented by campesterol, stigmasterol, sitosterol, and their common precursor squalene in the wax fraction of duckweed is rather surprising. As recently reported by Appenroth et al. (2017), phytosterols represent a significant part, 5%, of the total lipids in *Wolffia*, another duckweed representative. This is one of the highest fractions found in vegetative oils (Phillips et al., 2002), indicating these compounds potentially have important roles in the Lemnaceae family. Phytosterols, isoprenoid-derived molecules linked with fatty acid carbon chains of varying length, have been associated with the response to various abiotic stresses and non-host resistance to bacterial pathogens. For example, squalene is quite abundant in human skin where, together with free fatty acids, it represents the first line of defense against solar radiation by directly absorbing UV radiation, and it acts as an efficient scavenger for reactive molecular species generated by that radiation (De Luca and Valacchi, 2010).

In the turions, fully-saturated C24:0 was revealed as the most dominant fatty acid in the cuticle (Figure 5). Polyunsaturated linolenic fatty acid is a rather unusual component in plant cuticle. This class of fatty acids is more characteristically found among storage lipids and/or for membrane lipids. Evidence shows there is polyunsaturated C18:3 fatty acids present in plant surface structures: Hernández-Pinzón et al. (1999) indicated the abundance of these molecules in the polar soluble lipid fraction of the pollen coat (68.2%), and it was shown that this compound was relocated to the pollen surface during tapetum programmed cell death from the elaioplasts (Wu et al., 1997).

Among the cutin monomers in the duckweed fronds, a substantial amount was C18:3 (α-linolenic acid), but almost 15% was fully saturated longer-chain (24C) fatty acids and hydroxyl-fatty acids (Figures 4C,D). In the turion cutin, the most abundant fatty acids were C18:2 and fully-saturated C16:0. Though polyunsaturated fatty acids are not often found among cutin monomers, small amounts were detected in *Arabidopsis thaliana* (Franke et al., 2005; Fich et al., 2016). The major part of the fatty acids among the suberin monomers coincides with those in the cutin monomers (Graça, 2015). Cinnamic acid is an usual aromatic monomer of the polyaromatic domain in suberin and lignin (Bernards et al., 1995). Its detection in duckweed cutin could indicate suberized layers in addition to the cuticle.

Lignin-like and suberin-like substances are among of most native fluorophores in plant tissues (Donaldson and Williams, 2018). In duckweed fronds, we found that autofluorescence indicated the presence of polyaromatic structures such as suberin or lignin in the epidermal layer of the water-facing side (Figure 6). These types of substances are common in plant roots (Wilson and Peterson, 1983). The water-facing side of the duckweed fronds performs root-specific functions; therefore, it is not surprising that the chemical composition of this frond surface shows similarity to that found at the root surface.

Using cryo-SEM, we observed wax crystals on the regular backbone film structure of the *Spirodela* cuticle surface (Figure 2). The cuticle film on the adaxial side of the frond is most probably composed of fatty acids, the dominant component of duckweed waxes. We speculate that the wax crystals are composed of fatty alcohols, giving their ability to form surface crystals (Koch et al., 2006). After all, fatty alcohols were the second-most abundant chemical group in the wax, following fatty acids. The barely-distinguished nanoridge net on the native adaxial side of the fronds became profoundly visible on SEM images of samples treated with chloroform to remove the wax. On the contrary, at the abaxial side of the fronds emerged in water, SEM revealed a well-structured net-like structure covering the epidermal cells (Figure 2).

Similarly, an even more profound structure was detected on the abaxial side of *Spirodela* turions (Figure 3). To the best of our knowledge, for neither fronds nor turions can this net-like structure be assigned to a previously classified type of epicuticle “superimposed” wax structure. Following treatment with chloroform, this net remains intact. The chemical nature of the net components remains unknown and the subject of future investigations.

In summary, this study elucidated the structural and biochemical compositions of the cuticle in duckweed, *S. polyrhiza*. This is the first data published of this type, to the best of our knowledge, representing aquatic plants.

Microscopic investigations of the duckweed surfaces (cryo-SEM and fluorescence microscopy) uncovered significant differences in structures between the abaxial (water-facing) and adaxial (air-facing) sides of the plant's fronds. While the adaxial side, with multiple stomata complexes, is a smooth continuous sheet with randomly-distributed wax crystals, a rather typical structure for terrestrial species; the abaxial side is built from large oval cells covered with a distinct net-like structure resistant to chloroform and possessing autofluorescence characteristics of suberin and/or lignin.

Analysis using GC-MS of chloroform-extracted cuticle revealed a unique biochemical composition of cuticular waxes primarily identified via GC-MS/GC-FID as fatty acids and phytosterols (up to 60%). This high phytosterol content raises special interest because of their functions in the absorption of UV irradiation and scavenging of UV-generated radicals (De Luca and Valacchi, 2010) in addition to their potential in lowering human serum cholesterol (Kritchevsky and Chen, 2005).

Because the genome sequence of *S. polyrhiza* (Wang et al., 2014; Michael et al., 2017) is available, duckweed could be used as a model for studying fundamental processes related to plant protection against UV radiation and other environmental stresses. It could also be a promising platform for producing valuable products such as phytosterols.

## Author contributions

Conceived and designed the experiments: NB and JS. Performed the experiments: JS, AP, JL, GQ, QL, LS, GC, OK and YZ. Analyzed the data: JS, AP, and NB. Contributed reagents, materials, and analysis tools: JS, YZ and NB. Wrote the paper: NB, AP and JS.

### Conflict of interest statement

The authors declare that the research was conducted in the absence of any commercial or financial relationships that could be construed as a potential conflict of interest. The reviewer UH and handling Editor declared their shared affiliation.
