# Supplementary material for: Structural and Biochemical Properties of Duckweed Surface Cuticle
Source: Front Chem. 2018 Jul 26;6:317. doi: 10.3389/fchem.2018.00317 (PMC6070633; doi:10.3389/fchem.2018.00317)
Supplement: Supplementary file 2 [file Table_2.PDF]

Table 2. Grouping of duckweed ecotypes g, k, m, n, based on General Linear Model Multivariate analysis of cutin monomers content

#	Substance	GLM Multivariate tests significance of effects of duckweed ecotypes on individual substance content in cutin	GLM Post Hok Tukey HSD multiple comparison grouping			
			number of subsets of ecotypes	Homogeneous Subsets:		
				Subset 1	Subset 2	Subset 3
1	C16:0 FA	0.0014	3	g, m	g, n	k, n
2	C17:0 FA	0.1209	1	g, k, m, n		
3	C18:2 FA	0.0000	2	g	k, m, n	
4	C18:3 FA	0.0000	3	g, n	k	m
5	C18:0 FA	0.5526	1	g, k, m, n		
6	C20:0 FA	0.0001	2	g, k	m, n	
7	C22:0 FA	0.0001	2	g, k	m, n	
8	C23:0 FA	0.0031	2	g, k, m	k, n	
9	C24:0 FA	0.0288	2	g, k, m	k, m, n	
10	C25:0 FA	0.0002	3	g, k	k, n	m
11	C28:0 FA	0.0027	2	g, k	k, m, n	
12	C26:0 ω-HFA	0.0256	2	g, k, m	g, k, n	
13	C28:0 ω-HFA	0.0000	3	g	k, m	k, n
14	C30:0 ω-HFA	0.0000	3	g	k, n	m
15	C28:0 DFA	0.0000	2	g, m	k, n	
16	C16:0 2HFA	0.0008	2	g, k, m	n	
17	C20:0 2HFA	0.0000	2	g, k, m	n	
18	C22:0 2HFA	0.2982	1	g, k, m, n		
19	C23:0 2HFA	0.5076	1	g, k, m, n		
20	C24:0 2HFA	0.0051	2	g, k, m	n	
21	C25:0 2HFA	0.0014	2	g, k, n	m	
22	C26:0 2HFA	0.0003	2	g, k, m	n	
23	Cinnamic acid	0.0004	2	g, k, n	m	
