# Supplementary material for: Structural and Biochemical Properties of Duckweed Surface Cuticle
Source: Front Chem. 2018 Jul 26;6:317. doi: 10.3389/fchem.2018.00317 (PMC6070633; doi:10.3389/fchem.2018.00317)
Supplement: Supplementary file 3 [file Image_1.PDF]

Supplement Figure 1. Alignments of chloroplast DNA barcodes from *Spirodela polyrhiza* ecotypes G, K, M and N.

1A. Alignment of the chloroplast intergenic spacers *atpF-atpH* (ATP) sequences.
1B. Alignment of the chloroplast intergenic spacers *psbK-psbL* (PSB).

Supplement Figure 1A

```
G-ATP   caccaagtactacgcttagatttattggatttgttgctaaaatatcggtattaaacccaa
K-ATP   caccaagtactacgcttagatttattggatttgttgctaaaatatcggtattaaacccaa
M-ATP   caccaagtactacgcttagatttattggatttgttgctaaaatatcggtattaaacccaa
N-ATP   caccaagtactacgcttagatttattggatttgttgctaaaatatcggtattaaacccaa
        ************************************************************

G-ATP   aacccccggcggatggccagtggcccaaggaaacaaaagaatcagttacatttttcatat
K-ATP   aacccccggcggatggccagtggcccaaggaaacaaaagaatcagttacatttttcatat
M-ATP   aacccccggcggatggccagtggcccaaggaaacaaaagaatcagttacatttttcatat
N-ATP   aacccccggcggatggccagtggcccaaggaaacaaaagaatcagttacatttttcatat
        ************************************************************

G-ATP   actctcctcttatagataggactaacaaagaacagagttctttttgtatcacttcgcccc
K-ATP   actctcctcttatagataggactaacaaagaacagagttctttttgtatcacttcgcccc
M-ATP   actctcctcttatagataggactaacaaagaacagagttctttttgtatcacttcgcccc
N-ATP   actctcctcttatagataggactaacaaagaacagagttctttttgtatcacttcgcccc
        ************************************************************

G-ATP   ctttttttggttgatttcttttttttttatgggattttttaatggaataaattaaatcaa
K-ATP   ctttttttggttgatttcttttttttttatgggattttttaatggaatagattaaatcaa
M-ATP   ctttttttggttgatttcttttttttttatgggattttttaatggaatagattaaatcaa
N-ATP   ctttttttggttgatttcttttttttttatgggattttttaatggaatagattaaatcaa
        *************************************************.**********

G-ATP   ttaatttaattgaaaactttttaatttataattttattctaattaaattaaagtttacaa
K-ATP   ttaatttaattgagaactttttaatttataattttattctaattaaattaaagtttacaa
M-ATP   ttaatttaattgagaactttttaatttataattttattctaattaaattaaagtttacaa
N-ATP   ttaatttaattgagaactttttaatttataattttattctaattaaattaaagtttacaa
        *************.**********************************************

G-ATP   gaaaatacttattgggttaggtcctgggtattttgtcaattgataaataccttgtttgtt
K-ATP   gaaaatacttattgggttaggtcctgggtattttgtcaattgataaataccttgtttgtt
M-ATP   gaagatacttattgggttaggtcctgggtattttgtcaattgataaataccttgtttgtt
N-ATP   gaagatacttattgggttaggtcctgggtattttgtcaattgataaataccttgtttgtt
        ***.********************************************************

G-ATP   gcgttgcaacgcatacaaaaaaaggtttccgttacattatactaaaaactaaaaacggga
K-ATP   gcgttgcaacgcatacaaaaaaaggtttccgttactttatactaagaactaaaaacggga
M-ATP   gcgttgcaacgcatacaaaaaaaggtttccgttacattatactaagaactaaaaacggga
N-ATP   gcgttgcaacgcatacaaaaaaaggtttccgttacattatactaagaactaaaaacggga
        *********************************** *********.**************
G-ATP   aggaaaaaagcgagaggatctgctaattactaatcctaaaatcagtccttcccggaggta
K-ATP   aggaagaaagcgagaggatctgctaattactaatcctaaaatcagtccttcccggaggta
M-ATP   aggaagaaagcgagaggatctgctaattactaatcctaaaatcagtccttcccggaggta
N-ATP   aggaagaaagcgagaggatctgctaattactaatcctaaaatcagtccttcccggaggta
        *****.******************************************************
```

```
G-ATP   ttctctcaacgaataagtaattgttaaagtgcaatgttgatataattcgaaaaaacaaaa
K-ATP   ttctctcaacgaataagtaattgttagagtgcaatgttgatataattcgaagaaacaaaa
M-ATP   ttctctcaacgaataagtaattgttagagtgcaatgttgatataattcgaagaaacaaaa
N-ATP   ttctctcaacgaataagtaattgttagagtgcaatgttgatataattcgaagaaacaaaa
        **************************.************************.********

G-ATP   agcaagtctaagtcaaaaaaaattacgtacgtacttttatttcttctaaaattaaattaa
K-ATP   agcaagtctaagtcaaaaaaaattacgtacgtacttttatttcttctaaaattaaattaa
M-ATP   agcaagtctaagtcaaaaaaaattacgtacgtacttttatttcttctaaaattaaattaa
N-ATP   agcaagtctaagtcaaaaaaaattacgtacgtacttttatttcttctaaaattaaattaa
        ************************************************************

G-ATP   acaaatggattcgcaaataaaagtgctaatgccacaaccagtccataaattgttaaagct
K-ATP   acaaatggattcgcaaataaaagtgctaatgccacaaccagtccataaattgttaaagct
M-ATP   acaaatggattcgcaaataaaagtgctaatgccacaaccagtccataaattgttaaagct
N-ATP   acaaatggattcgcaaataaaagtgctaatgccacaaccagtccataaattgttaaagct
        ************************************************************
```

Supplement Figure 1B

```
G-PSB   ttcttatactctactgaaaaattcatgatttttttgataaaaaaggattcttatatctaa
N-PSB   ttcttatactctactgaaaaattcatgatttttttgataaaaaaggattcttatatctaa
K-PSB   ttcttatactctactgaaaaattcatgatttttttgat-aaaaaggattcttatatctaa
M-PSB   ttcttatactctactgaaaaattcatgatttttttgat-aaaaaggattcttatatctaa
        ************************************** *********************

G-PSB   taataattgataacataatatgagtcttagcttataaatataaatcctcattagaaaaga
N-PSB   taataattgataacataatatgagtcttagcttataaatataaatcctcattagaaaaga
K-PSB   taataattgataacataatatgagtcttagcttataaatataaatcctcattagaaaaga
M-PSB   taataattgataacataatatgagtcttagcttataaatataaatcctcattagaaaaga
        ************************************************************

G-PSB   aataaatttaaattcttgtatattggataaaaggagcgataagtttggatcagtcaattt
N-PSB   aataaatttaaattcttgtatattggataaaaggagcgataagtttggatcagtcaattt
K-PSB   aataaatttaaattcttgtatattggataaaaggagcgataagtttggatcagtcaattt
M-PSB   aataaatttaaattcttgtatattggataaaaggagcgataagtttggatcagtcaattt
        ************************************************************

G-PSB   ccccgttccgtacgctcttccagtgagcaagtactttctttattagcttatgtttttcca
N-PSB   ccccgttccgtacgctcttccagtgagcaagtactttctttattagcttatgtttttcca
K-PSB   ccccgttccgtacgctcttccagtgagcaagtactttctttattagcttatgtttttcca
M-PSB   ccccgttccgtacgctcttccagtgagcaagtactttctttattagcttatgtttttcca
        ************************************************************

G-PSB   caatactttattgttaatattagaataaccattttggtaacgaacaaatcataatcttaa
N-PSB   caatactttattgttaatattagaataaccattttggtaacgaacaaatcataatcttaa
K-PSB   caatactttattgttaatattagaataaccattttggtaacgaacaaatcataatcttaa
M-PSB   caatactttattgttaatattagaataaccattttggtaacgaacaaatcataatcttaa
        ************************************************************

G-PSB   tttcagaaaaaaattcatgaatttgaaaattcagtttttttagaaaaaaacacttaatta
N-PSB   tttcagaaaaaaattcatgaatttgaaaattcagtttttttagaaaaaaacacttaatta
K-PSB   tttcagaaaaaaattcatgaatttgaaaattcagtttttttagaaaaaaacacttaatta
M-PSB   tttcagaaaaaaattcatgaatttgaaaattcagtttttttagaaaaaaacacttaatta
        ************************************************************

G-PSB   aaaaaatttgttctttattttttcatattttttttttttgtttttggggcatgtcaaaat
N-PSB   aaaaaatttgttctttattttttcatattttttttttttgtttttggggcatgtcaaaat
K-PSB   aaaaaatttgttctttattttttcatattttttttttttgtttttggggcatgtcaaaat
M-PSB   aaaaaatttgttctttattttttcatattttttttttttgtttttggggcatgtcaaaat
        ************************************************************

G-PSB   aatacatgtgttacataactcaaatggataatctattcccttttaccccaaaaatgatcc
N-PSB   aatacatgtgttacataactcaaatggataatctattcccttttaccccaaaaatgatcc
K-PSB   aatacatgtgttacataactcaaatggataatctattcccttttaccccaaaaatgatcc
M-PSB   aatacatgtgttacataactcaaatggataatctattcccttttaccccaaaaatgatcc
        ************************************************************

G-PSB   tatcttggagattgtgtaatgcttactctcaaactt
N-PSB   tatcttggagattgtgtaatgcttactctcaaactt
K-PSB   tatcttggagattgtgtaatgcttactctcaaactt
M-PSB   tatcttggagattgtgtaatgcttactctcaaactt
        ************************************
```
