# Supplementary material for: Structural and Biochemical Properties of Duckweed Surface Cuticle
Source: Front Chem. 2018 Jul 26;6:317. doi: 10.3389/fchem.2018.00317 (PMC6070633; doi:10.3389/fchem.2018.00317)

Supplement Figure 2. The chemical composition of duckweed fronds cuticle as revealed by GS-MS and GS-FID analysis. Extraction time – 30 seconds. (A) Components percentage in wax fraction of the duckweed fronds surface. (B) Percentage of individual components in the wax fraction of duckweed fronds surface. C16:0 FA - hexadecanoic acid; C18:2 FA - (9Z,12Z)-octadeca-9,12-dienoic acid; C18:3 FA - octadecatrienoic acid; C18:0 FA - octadecanoic acid; C20:0 FA - icosanoic acid; C22:0 FA - docosanoic acid; C24:0 FA - tetracosanoic acid; C26:0 FA - hexacosanoic acid; C30:0 FA - triacontanoic acid; C22:0 OL - docosan-1-ol; C24:0 OL - tetracosan-1-ol; C26:0 OL - hexacosan-1-ol; UI – unidentified substances.

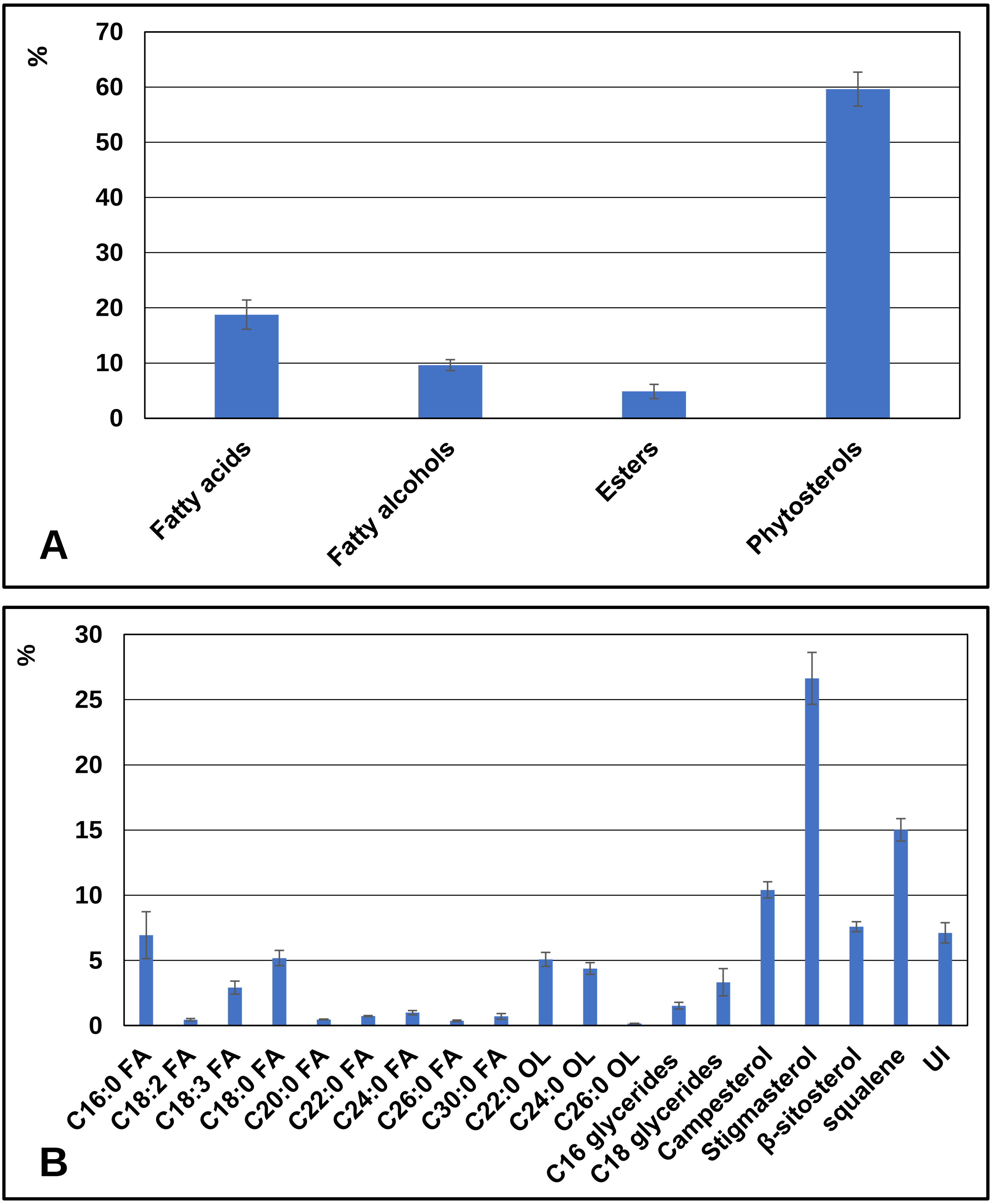

Supplement: Supplementary file 4 [file Image_2.PDF]
